# An Account of Scurvy, as It Has Recently Prevailed in Some Parts of the Russian Territory

**Published:** 1826-06

**Authors:** 


### Art. IV.—*An Account of Scurvy, as it has recently prevailed in some Parts of the Russian Territory.* By — Lee, m.d.

Having been engaged, during a year's residence in the Crimea and in the countries on the northern shores of the Black Sea, in collecting information respecting the climate and diseases of these extensive regions, I was much astonished to find that a species of land-scurvy had been prevailing extensively, not only in the hospitals of Nicolaef, Cherson, and Sevastopole, but in the military colonies established from the Dnieper to the Bessambia. In the period of its occurrence, and in many of the leading symptoms, it forcibly recalled to my mind the discussions which took place before a committee of the House of Commons on the scurvy which attacked those confined in the great Penitentiary two years ago. It is also a singular circumstance, mentioned by a Russian medical author, that, in 1786, at the siege of Azoff, a similar malady broke out, and committed great ravages in the Russian army; and subsequently to this period, during their march to Ockcihoff, though their diet did not consist of salted provisions.

It is necessary to state, before giving any account of the particular symptoms of the disease, that the medical officers of the different hospitals are accustomed to use the term *scurvy* in a more vague and extensive sense than we usually do in England: they apply it to many obscure cases of visceral derangement and cachectic states of the system, with spongy and swelled gums; and therefore, in looking over the registers of the hospitals, it appears that some of these are constantly present under the head of scurvy. There can be no doubt, however, that the disease had actually commenced in April 1823; the proportion of these cases with spongy gums being much larger than usual, but no cases of severity were then observed; and it was not until February 1824, that the disease appeared in its perfect form. Then most of the patients had the surface of the body covered, to a lesser or greater extent, with petechiæ or vibices; the gums were spongy, and often copious hemorrhages took place from them; the teeth became loose; the breath extremely fetid; and the countenance was sallow and bloated, and precisely similar to those afflicted with anasarca. Sometimes the inferior extremities were almost entirely covered with ecchymoses, or livid spots; but these were rarely observed on the arms or trunk of the body. Petechiæ not unfrequently appeared all over the surface. Violent pains occurred in the limbs during the night, so as to deprive the patients of sleep; and the strongest narcotic medicines had no effect in alleviating the sufferings. A frequent occurrence in this complaint was the loss of the use of the limbs and contractions of the tendons of the hams. In several, this continued for three or four months, the patients being confined constantly to bed, and unable even to walk upon crutches; yet all these recovered ultimately.

It does not appear that any affection of the mucous membrane of the intestines existed, as diarrhœa or dysentery did not generally occur.

Slight œdematous swellings of the lower extremities occasionally were witnessed. There was commonly, before the appearance of these symptoms, an unusual feeling of lassitude and depression of spirits. There was no thirst, and the appetite continued good in many during the whole course of the disease.

In the hospital at Nicolaef, only one case proved fatal, and this rather from a dose of calomel, which brought on salivation and diarrhœa, than from the scurvy.

While this disease was prevailing, the buboes in a syphilitic ward of the hospital put on the scorbutic appearance. The skin, for several inches around, became black or livid, like the ecchymoses on the legs in other cases. Hemorrhagy in no case took place from these buboes. Other symptoms of scurvy manifested themselves in those affected with syphilis, and in some dropsical patients. The common ulcers in the hospital entirely escaped the disease.

Two cases of pneumonia were brought into the hospital, and, though not bled profusely, they both speedily sunk. Many cases of pneumonia occurred in the military colonies, and all that were bled died. Afterwards large doses of emetic tartar were given with success.

It was observed that the disease was more frequent and severe among the convicts, and others under the influence of the depressing passions, than among the other seamen.

Although this disease seldom proved fatal in these great naval hospitals, yet Mr. Prout was informed that many persons, particularly women, died of it in the great villages belonging to the crown, beyond the Dnieper.

In the cases of ophthalmia, there was usually a black areola around the eye, and the vessels of the conjunctiva, instead of carrying a florid blood, were gorged with blood of a dark venous colour.

The treatment consisted chiefly in the internal use of mineral acids, vinegar, and bitters; and externally of fomentations with aromatic plants, and vinegar, to the legs. Citric acid is not to be found in this country.

I ought to have mentioned that this disease occurred not only in these hospitals, and in the military colonies, but in the Crimea, and in the governments of Cherson and Agehaterinorloff; in a word, in all that tract of country which had suffered from the visitations of the locusts. These formidable insects appeared first in the Crimea, in the summer of 1820, in small numbers, and are supposed to have crossed the narrow strait of the Bosphorus from the island of Taman. At first they did little injury; and even the following summer many doubted if they were the true locust. In the summer of 1822, a season of great heat and drought, they appeared in vast numbers, not only in the Crimea, but all along the shores of the Black Sea. A prodigious army of them crossed the Danube, and ravaged Moldavia. In 1823, they appeared in still greater numbers, and devoured every thing of a vegetable nature that came in their way. It was precisely at this period that the disease commenced, and there can be little doubt that it ought to be attributed to a want of the usual supply of the sour crout and prepared cabbages, and other vegetables, for the winter. Every peasant in this country lays in an ample stock of these for the winter; but all green vegetables had attained an unusual price at this period, and it was in the power of few to make the usual provision.

In all the hospitals the patients were supplied, as they usually are, with fresh animal food, bread, and grits for gruel; yet the disease was not arrested.

A short account of the climate of the Crimea, and of the country lying along the northern shores of the Black Sea, may not be wholly without interest to those engaged in the improvement of medical topography. The impossibility of determining the precise nature of the climate of a country from its geographical position alone, is very manifest from what we witness in the Crimea. The southern coast, which is protected by the range of mountains running from west to east, enjoys one of the most mild and beautiful climates of Europe, being equally removed from those extremes of heat and cold which prevail in the northern side of these mountains, and in all that extensive country between the Danube and the Don. Along this sheltered border of the Crimea, the vine, olive, pomegranate, and other plants of southern Europe, grow luxuriantly in the open air. The thermometer rarely falls below zero *(of Reaumur?)* and the snow never remains upon the ground more than a few days, during the whole course of the year.

At Synpheropole, a distance of not more than forty English miles on the other side, the thermometer has been seen as low as 23° of Reaumur below the freezing point, and the winters are usually extremely severe. In the imperial garden of Nehiter, on the coast, the thermometer rarely mounts above 25° of Reaumur; while in the interior it frequently reaches thirty, and remains only a degree or two below this for many successive days. Yet, even upon the sea-shore, and still more in the interior, the changes of temperature are sudden and violent in the highest degree; and so well aware are Tartars of this, that they rarely quit home, even in the finest weather, without a thick mantle or cloak.

In various parts of the Crimea, along the course of some of the rivers, and at Kosloff and near the isthmus of Perecop, there are marshes which emit exhalations of the most pernicious character. Fever, intermittent and remittent, and often of a bad form, is frequent in these districts; and, in particular seasons, the whole population appears to suffer. The pallid, sallow complexion of the people constantly reminded me of the appearance of those who live in the marshy districts on the shores of the Mediterranean, in Italy and France.

This autumn (1825) the affection of the brain was unusually severe, and, in spite of the free detraction of blood, ended in effusion into the ventricles in several instances which came under my notice.

